# Membrane Targeting of Disheveled Can Bypass the Need for Arrow/LRP5

**DOI:** 10.1038/s41598-017-04414-0

**Published:** 2017-07-31

**Authors:** Prameet Kaur, Vanessa Yuk Man Lam, Anirudh Gautam Mannava, Jahnavi Suresh, Andreas Jenny, Nicholas S. Tolwinski

**Affiliations:** 1 Yale-NUS College, National University of Singapore, Block MD6, Centre for Translational Medicine, Yong Loo Lin School of Medicine, 14 Medical Drive, Level 10 South, 10-02M, Singapore, 117599 Singapore; 2 Department of Biological Sciences, National University of Singapore, Block MD6, Centre for Translational Medicine, Yong Loo Lin School of Medicine, 14 Medical Drive, Level 10 South, 10-02M, Singapore, 117599 Singapore; 3 Department of Developmental and Molecular Biology, Department of Genetics, Albert Einstein College of Medicine, 1300 Morris Park Ave., Bronx, NY 10461 USA

## Abstract

The highly conserved Wnt signaling pathway regulates cell proliferation and differentiation in vertebrates and invertebrates. Upon binding of a Wnt ligand to a receptor of the Fz family, Disheveled (Dsh/Dvl) transduces the signal during canonical and non-canonical Wnt signaling. The specific details of how this process occurs have proven difficult to study, especially as Dsh appears to function as a switch between different branches of Wnt signaling. Here we focus on the membrane-proximal events that occur once Dsh is recruited to the membrane. We show that membrane-tethering of the Dsh protein is sufficient to induce canonical Wnt signaling activation even in the absence of the Wnt co-receptor Arrow/LRP5/6. We map the protein domains required for pathway activation in membrane tethered constructs finding that both the DEP and PDZ domains are dispensable for canonical signaling only in membrane-tethered Dsh, but not in untethered/normal Dsh. These data lead to a signal activation model, where Arrow is required to localize Dsh to the membrane during canonical Wnt signaling placing Dsh downstream of Arrow.

## Introduction

Wnt signaling consists of a series of evolutionarily conserved pathways taking part in many developmental processes^1–3^. The main or canonical signaling branch regulates cytoplasmic levels of Armadillo (Arm, β-catenin) affecting cell fate and proliferation^3^. Non-canonical pathways are involved in a variety of cellular polarity processes from convergence & extension in vertebrate gastrulation to ommatidial rotation in the Drosophila eye^4–7^. In canonical signaling, Disheveled (Dsh in Drosophila, Dvl in vertebrates) functions to relay the Wnt message from the Wnt receptors, Frizzled (Fz) and Arrow (Arr in Drosophila, LRP-5/6 in vertebrates), nucleating the membrane-proximal activation complex (signalosome)^8^. Dsh, as the most downstream shared component between canonical and non-canonical signaling likely also determines which pathway is activated^9, 10^.

The canonical Wnt signaling pathway is activated by Wnt binding to its receptors, Fz and Arr. This tri-partite complex transmits the extracellular signal to the intracellular components^11^ by recruiting Dsh to the membrane and forming the membrane-proximal activation complex consisting of Arr, Axin, and the kinases CK1 and GSK3^8^. Once this complex forms, the cytoplasmic destruction complex, consisting of APC, Axin, CK1 and GSK3 is disrupted allowing Arm to escape phosphorylation and ubiquitin mediated degradation by the proteasome. As the destruction complex ceases to do its work, levels of Arm increase, and Arm enters the nucleus where, along with the transcription factor TCF, it activates transcription of target genes^12–16^.

When Arr was originally discovered as a co-receptor for Fz, it seemed relatively obvious that it should function upstream of Dsh as most intracellular signaling components function downstream of transmembrane ligand receptors. This turned out, however, not to be the case as Arr was shown to function downstream of Dsh^17–22^. This discovery led to the current model of activation complex assembly, where the Fz receptor recruits Dsh to the membrane forming a binding site for other pathway components, and bringing the cytoplasmic, C-terminal portion of Arr into close proximity with GSK3 and CK1 leading to Arr phosphorylation. Phosphorylated Arr/Lrp in turn becomes a binding site for Axin, the limiting factor for the assembly of the destruction complex, functionally taking the destruction complex apart and preventing Arm degradation^18, 23–27^. Phosphorylated Arr/Lrp also directly inhibits GSK3 by providing pseudo-substrates for GSK3 to bind^28, 29^.

The Dsh protein contains highly conserved DIX, PDZ and DEP domains (Sup. Figure 1). The PDZ and DIX domains are thought to be involved in canonical signaling where the PDZ domain interacts with the intracellular domain of Fz^30^, and the DIX domain binds tightly to the DAX (also called DIX) domain of Axin^31^. The DEP domain was originally thought to be specific to non-canonical signaling as the original planar cell polarity defect causing mutation *dsh* ^1^ contained a point mutation in the DEP domain that was thought to prevent Dsh protein from localizing to the membrane^32^. Structurally the DEP domain stabilizes Dsh’s interaction with the membrane by binding to charged phospholipids at the plasma membrane^33, 34^, and binding directly to Fz^35, 36^. Additionally, the DEP domain was recently shown to function in canonical signaling by nucleating signalosome assembly^36, 37^. Taken together, the function of the DEP and PDZ domains is to localize Dsh to the membrane through Fz. The DIX and DEP domains then can function to nucleate signalsomes^31, 36–38^.

Despite Dsh being discovered more than 20 years ago as a Wnt pathway component^39^, the mechanism of how it relays signal to specific cellular responses is still poorly understood^40^. Here we investigate the role of plasma membrane localization of Dsh protein. We utilize the *Drosophila* embryo to express an allelic series of Dsh proteins in both *dsh* and *arr* null genetic backgrounds. We use stringent developmental rescue and molecular assays to establish the functionality of Dsh alleles. We find that Arr is required for canonical pathway activation only if Dsh is not membrane localized. We find that both PDZ and DEP domains are dispensable for signaling when Dsh is membrane localized, but not when it is cytoplasmic.

## Results

### Expression of membrane-tethered Dsh activates signaling

**Figure 1:**
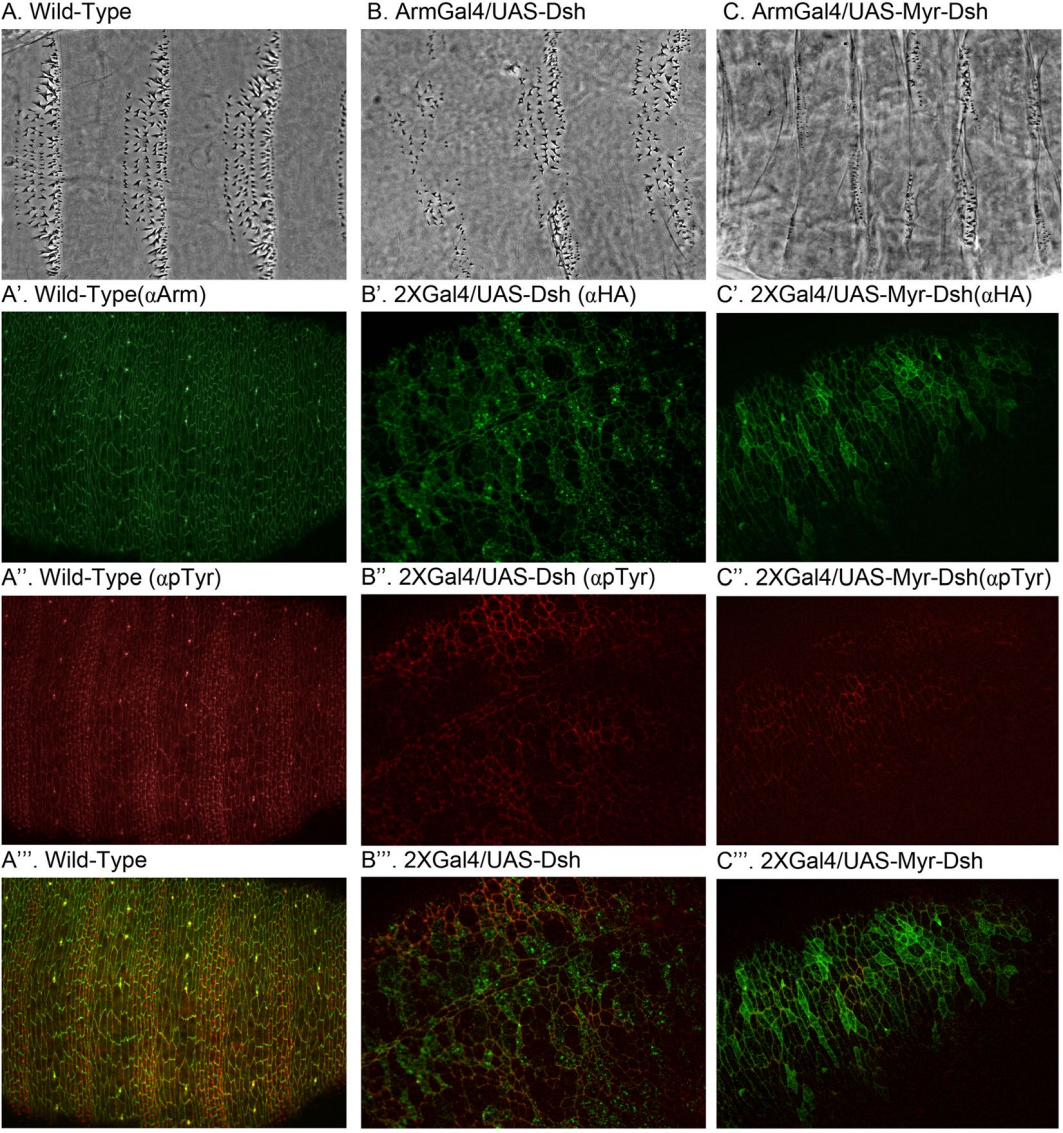
Cuticle preparations of (**A**) Wild type embryo showing six rows of denticles per segment. (**B**) Embryo expressing Dsh resulting in expansion of naked cuticle causing loss of one or two rows of denticles. (**C**) wild type embryo expressing Myr-Dsh showing signaling activation phenotype (GOF). To increase expression levels, we doubled embryonic Gal4 by combining daGal4 and ArmGal4 into a 2XGal4 line. (A’-A”) Wild-type embryo stained for Arm and denticle precursors (pTyr). (B’-B”) Embryo overexpressing Dsh stained for ectopic tag HA and denticle precursors (pTyr). Similar staining in wild type embryo expressing Myr-Dsh (C’-C”).”’ panels show merged images.

Although most studies have reported that Dsh is a cytoplasmic protein, there have been some reported instances where it was found in the nucleus^41^. In order to test the sufficiency of Dsh protein expression at the membrane, we attached a Src derived myristoylation (Myr) sequence to the N-terminus of Dsh. This sequence was originally used to tether Arm protein to the membrane, and we have found it highly effective for membrane localization of GSK3, Axin and APC^16, 42–44^. We proceeded to express tethered and un-tethered Dsh versions in embryos to test their effect on patterning. Normal Drosophila embryos show a repeating pattern of naked cuticle and denticle covered cuticle a result of segment polarity patterning^45^. When Wnt signaling is turned off, all ventral epidermal cells produce denticles. The opposite is true when Wnt is turned on ectopically and most cells do not make denticles causing the ‘naked’ phenotype. When we expressed Dsh and Myr-Dsh in embryos, in both cases we saw a strong Wnt activation as visualized by a naked phenotype (Compare a control embryo (ArmGal4) in Fig. 1A with Fig. 1B,C respectively). Both Dsh isoforms were tagged with HA, so we could examine the localization in cells with anti-HA staining (Fig. 1B’,C’). Additionally, we used phospho-tyrosine staining to visualize cell outlines and denticle precursors (Fig. 1A”–C”). We observed a concentration of Myr-Dsh at the membrane, and a more diffuse, intracellular localization for Dsh with some punctate structures observed at higher expression conditions (Fig. 1B’).

**Figure 2:**
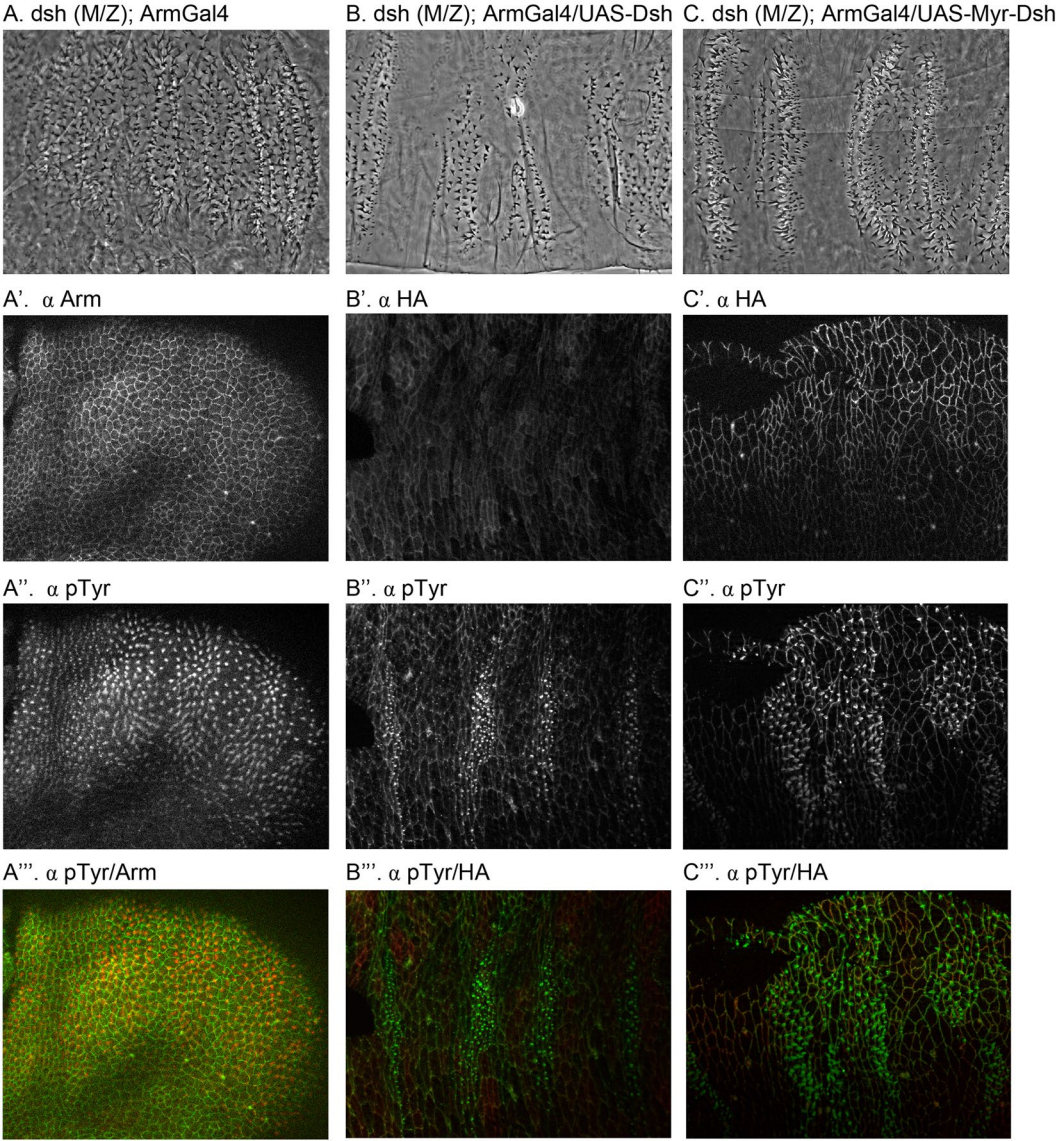
Cuticle preparations of (**A**) *dsh* (M/Z) mutant showing a lawn of denticles without activation of signaling. (**B**) *dsh* (M/Z) mutant embryo expressing Dsh resulting in expansion of naked cuticle. (**C**) *dsh* (M/Z) mutant embryo expressing Myr-Dsh showing signaling activation phenotype. (A’-A”) *dsh* (M/Z) mutant embryo stained for Arm and denticle precursors (pTyr). (B’-B”) *dsh* (M/Z) mutant embryo expressing Dsh stained for ectopic tag HA and denticle precursors (pTyr). Similar staining in *dsh* (M/Z) mutant embryo expressing Myr-Dsh (C’-C”).”’ panels show merged images.

Loss of Dsh leads to a strong segment polarity phenotype^46^. To establish the functionality of Dsh and Myr-Dsh, it was necessary to express these in a *dsh* null background. Therefore, we made maternal and zygotic *dsh* null embryos (*dsh* M/Z) completely lacking Dsh activity by crossing females with *dsh* germline clones^47^ (i.e. the only laid eggs are homozygous mutant) with males providing re-expressed UAS-Dsh and UAS-Myr-Dsh (note that paternally rescued embryos were excluded from analysis by being y^+^; see Materials and Methods). As expected, expression of Dsh rescues the canonical Wnt signaling defect of *dsh* mutants and causes some ectopic activation leading to a mild naked phenotype (Compare Fig. 2A and B). Similarly, Myr-Dsh rescues the loss of signaling as shown by the naked cuticle patches in these embryos (Fig. 2C). We visualized this both at the cuticle level, and used pTyr/HA staining to show loss of denticle precursors in Dsh expressing cells (Fig. 2B’,C’ and 2B”, C”, respectively). Taken together, these results show that at least for canonical Wnt activation, membrane localized Dsh is active and asks the question whether Dsh membrane localization is sufficient to bypass the requirement of Wnt co-receptors.

### Membrane tethered Dsh activates signaling in arr mutants

**Figure 3:**
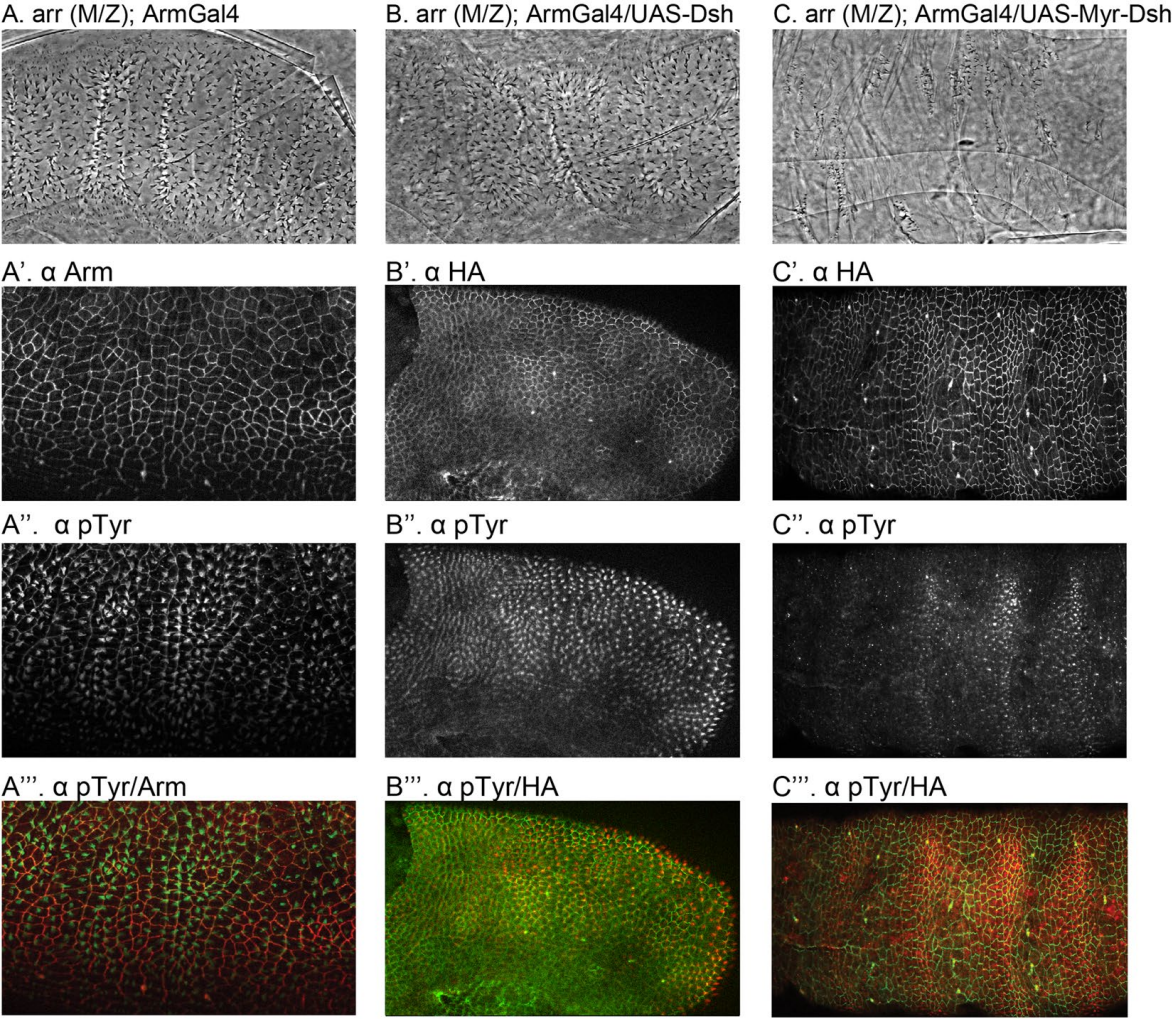
Cuticle of (**A**) *arr* (M/Z) mutant showing the wingless phenotype. (**B**) *arr* M/Z mutant expressing Dsh and showing a lawn of denticles without activation of signaling (**C**) *arr* (M/Z) mutant expressing Myr-Dsh showing an activation of signaling and thus a suppression of patterning defects. (A’-A”) *arr* (M/Z) mutant embryo stained for Arm and denticle precursors (pTyr). (B’-B”) *arr* (M/Z) mutant embryo expressing Dsh stained for ectopic tag HA and denticle precursors (pTyr). Similar staining in *arr* (M/Z) mutant embryo expressing Myr-Dsh (C’-C”).”’ panels show merged images.

The Wnt co-receptor Arr binds Wnts along with Fz^18, 19, 21^. In most signaling pathways, this should place it epistatically upstream of an intracellular component such as Dsh as is the case for Fz^48^. However, this is not the case for Arr and Dsh, as simple overexpression of Dsh in an *arr* mutant does not activate the pathway^17^. We repeated this experiment in order to compare the function of Myr-Dsh and Dsh, and as expected we find that expression of Dsh in maternally and zygotically mutant *arr* embryos does not activate signaling (Compare Fig. 3A to Fig. 3B). In contrast to normal Dsh, expression of membrane-tethered Myr-Dsh strongly activates signaling in *arr* (M/Z) embryos (Fig. 3C). Consistently, staining embryos for pTyr and HA-Dsh reveals denticle covered embryos in *arr* (M/Z); ArmGal4-UAS-Dsh despite the presence of HA-Dsh (Fig. 3B’,B”). However, we observed a loss of denticles in *arr* (M/Z) expressing Myr-Dsh (Fig. 3C’,C”). From these experiments, we conclude that signal activation through Dsh is indeed downstream of Arr, and that for the activation to occur, the requirement for Arr can be bypassed by localizing Dsh to the membrane.

### Dsh domains at the membrane

**Figure 4:**
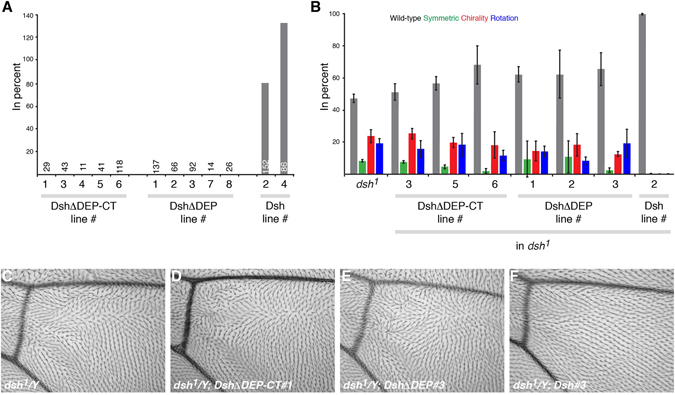
Variants of Dsh lacking the DEP domain and C-terminus (DshΔDEP-CT) or the DEP domain (DshΔDEP) expressed under the endogenous promoter do not rescue canonical Wnt or PCP signaling. (**A**) Relative rescue of lethality of indicated transgenic lines. In contrast to wild-type Dsh, no rescue of the *dsh* ^*V26*^ null allele is detected with any of the Dsh variants (five independent transgenic insertions tested for mutant Dsh forms). **B–F**) In contrast to wild-type Dsh, none of the Dsh variants is able to rescue the PCP defects of the PCP specific *dsh* ^*1*^ allele in the eye (**B**; wild-type, symmetric ommatidia, chirality and rotation defects were scored) or wing (**C–F** show enlargement of wing areas distal to the posterior cross veins). Three independent transgenic lines were assessed for rescue of PCP signaling.

**Figure 5:**
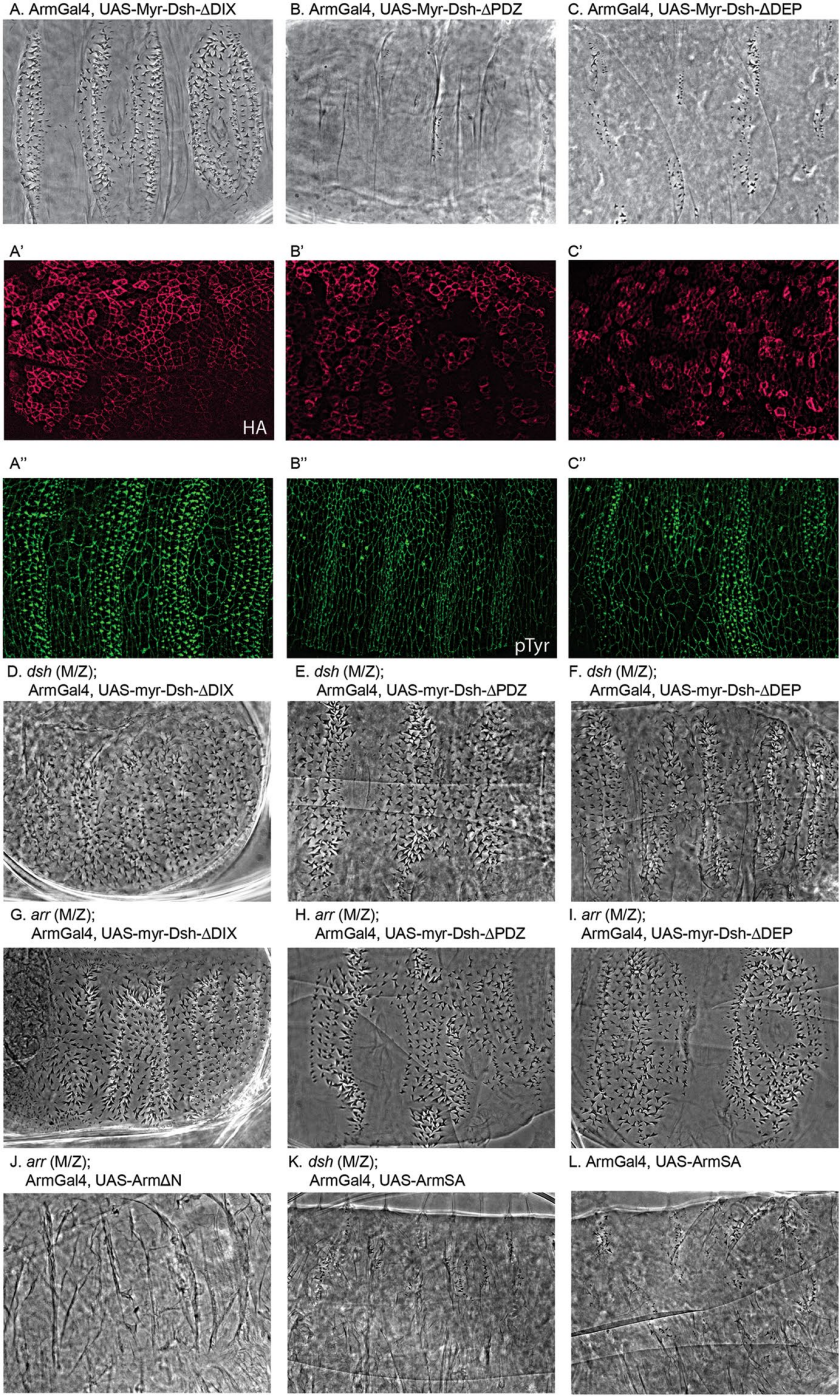
Cuticle of (**A**) Myr-DshΔDIX expressed in ‘wild type’ embryos, producing more ectopic denticles due to signaling inactivation. (**B**,**C**) Cuticles of Myr-DshΔPDZ and Myr-DshΔDEP expressing embryos, showing a naked phenotype (GOF). Staining for ectopic tag HA and denticle precursors (pTyr) in wild type embryos expressing Myr-DshΔDIX (A’-A”), Myr-DshΔPDZ (B’-B”) and Myr-DshΔDEP (C’-C”). **D–F**) Myr-DshΔPDZ (**E**) and Myr-DshΔDEP (**F**) expression in *dsh* (M/Z) mutant embryos rescued Wnt signaling whereas expressing Myr-DshΔDIX (**D**) in *dsh* (M/Z) mutant embryos did not. (**G**) Arrow null embryo expressing Myr-DshΔDIX showing no activation of signaling and hence the wingless phenotype. (**H**–**I**) Arrow null mutant suppressed by expression of Myr-DshΔPDZ and Myr-DshΔDEP suggesting requirement for a membrane recruitment. Cuticle of *arr* (M/Z) (**J**), *dsh* (M/Z) (**K**) and wildtype (**L**) embryos expressing activated Arm alleles either lacking the N-terminus (ArmΔN) or a specific ArmS56A substitution (ArmSA) both of which block phosphorylation and degradation of Arm. Expression in these backgrounds led to loss of denticles, reflecting constitutive activation of Wg signaling.

In order to analyze this result further, we determined the domain requirement of Dsh for activation of Wnt signaling by taking a structure function approach. Based on results with the *dsh* ^1^ allele causing PCP specific defects, the DEP domain was sometimes considered more important for non-canonical Wnt signaling than for canonical signaling, although overexpression and *in vitro* experiments suggested a role for the DEP domain also in canonical signaling^35, 36, 49, 50^. We therefore first verified that, compared to wild-type Dsh^51, 52^, Dsh lacking the DEP domain or the DEP domain and C-terminus expressed under control of its endogenous promoter cannot rescue the *dsh* ^*V26*^ null allele. Indeed, five independent transgenic insertions lacking the DEP or DEP-C-terminal domain each fail to rescue viability (canonical signaling; Fig. 4A). Similarly, the three lines tested also are unable to rescue the PCP defects of the *dsh* ^1^ allele in the eye and wing (Fig. 4B–F). We then investigated the effect of individual domains of Dsh in the membrane proximal activation complex. We made membrane tethered constructs where individual domains were deleted and expressed them in wild type embryos, *dsh* (M/Z) and *arr* (M/Z) mutant embryos (Sup. Figure 1). We find that expression of Myr-Dsh lacking either the PDZ or DEP domains can activate the pathway. Both proteins when expressed in otherwise wildtype embryos show a strong gain of function (GOF) phenotype leading to nearly naked patterning (Fig. 5B,C). In contrast, expression of the Myr-DshΔDIX leads to a strong loss of signaling phenotype with many ectopic denticles, thus likely acting as dominant negative (Fig. 5A). This was verified with HA/pTyr staining to show loss of denticle precursors in Dsh expressing cells (Fig. 5B’,C’ and 5B”, C”, respectively).

We then proceeded to test the deletion constructs (Sup. Fig. 1) in *dsh* (M/Z) loss of function mutants to assess their ability to rescue the loss of endogenous Dsh. As expected from the dominant negative effect displayed in a wild-type background, Myr-DshΔDIX failed to rescue signaling in embryos (Fig. 5D). Dsh lacking the DEP or PDZ domains rescued *dsh* (M/Z) embryos when expressed as Myr-DshΔDEP and Myr-DshΔPDZ (Fig. 5E,F; quantified in Table 1). This finding confirmed the notion that the DEP and PDZ domains stabilize Dsh membrane localization. Nevertheless, although with respect to frequency of rescued embryos, rescue was efficient, the extent of the rescue was weaker than with the full-length Myr-Dsh (Fig. 2, compare the amount of naked cuticle between conditions) suggesting that the DIX and PDZ domains may have some further function in signal transduction at the membrane.

**Table 1:** Quantification of embryonic phenotypes.

	ArmGal4	*dsh* (M/Z)	*arrow* (M/Z)
UAS-Dsh	Naked 100%	naked cuticle (rescue or GOF) 50%	*wg* phen. 100%
	N > 100	N = 98	N = 220
UAS-MyrDsh	Naked 100%	Naked 48%	Naked 49%
	N > 100	N = 95	N = 223
UAS-Myr-DshΔDIX	*wg* phen 100%	*wg* phen 98%	*wg* phen. 100%
	N > 100	N = 101	N = 288
UAS-Myr-DshΔPDZ	Naked cuticle expansion 100%	Naked 48%	Naked 49%
	N > 100	N = 120	N = 294
UAS-Myr-DshΔDEP	Naked cuticle expansion 100% N > 100	Naked 48% N = 154	Naked 47% N = 138

Column (ArmGal4) represents the phenotype of a simple Gal4 driver to UAS construct cross where all embryos are expected to overexpress Dsh. Column (*arr*) represents embryos where all maternal *arr* is removed, paternally rescued embryos are removed by GFP selection and 50% of embryos are expected to express Dsh. Therefore, ‘50% naked cuticle’ corresponds to full suppression. Column (*dsh*) represents embryos where all maternal endogenous Dsh is removed, paternally rescued embryos are ignored based on y + , and 50% of embryos are expected to express UAS-Dsh. Therefore, ‘50% naked cuticle’ corresponds to full rescue. The naked phenotype is defined as fewer denticles than in wild type, but not necessarily that all denticles are absent.

Next, we expressed the membrane tethered deletion constructs in *arr* mutant embryos. Again, we find that in contrast to Myr-Dsh, Myr-DshΔDIX failed to rescue signaling in embryos lacking both maternal and zygotic Arr (Fig. 5G). Expression of Myr-DshΔDEP and Myr-DshΔPDZ showed rescue in *arr* (M/Z) embryos as revealed by distinct regions of naked cuticle in *arr* mutants (Fig. 5H,I; quantified in Table 1). Taken together, these results suggest that bringing Dsh to the membrane can bypass the requirement for Arr, but that the DIX domain is required to activate signaling even under those circumstances, while both the DEP and PDZ are dispensable, but may enhance signaling as the rescue was not as good as with full-length Myr-Dsh.

Dsh forms the signalosome at the membrane to activate signaling by inhibiting the action of the destruction complex^40^. Downstream, Arm protein levels increase and signaling is activated. We tested whether signaling could be activated downstream of both complexes when disrupted. We expressed Arm alleles that were activated either by deleting the N-terminus or by changing a specific phosphorylation site (ArmS56A^16, 42, 53, 54^), both of which block phosphorylation and degradation of Arm. Their expression in *dsh* (M/Z), *arr* (M/Z) or wildtype embryos led to loss of denticles or an activation of Wnt signaling (Fig. 5J–L). These results suggest that the downstream pathway is unaffected by the loss of the signalosome (see also discussion).

### Role of membrane-tethered Dsh in canonical signaling

**Figure 6:**
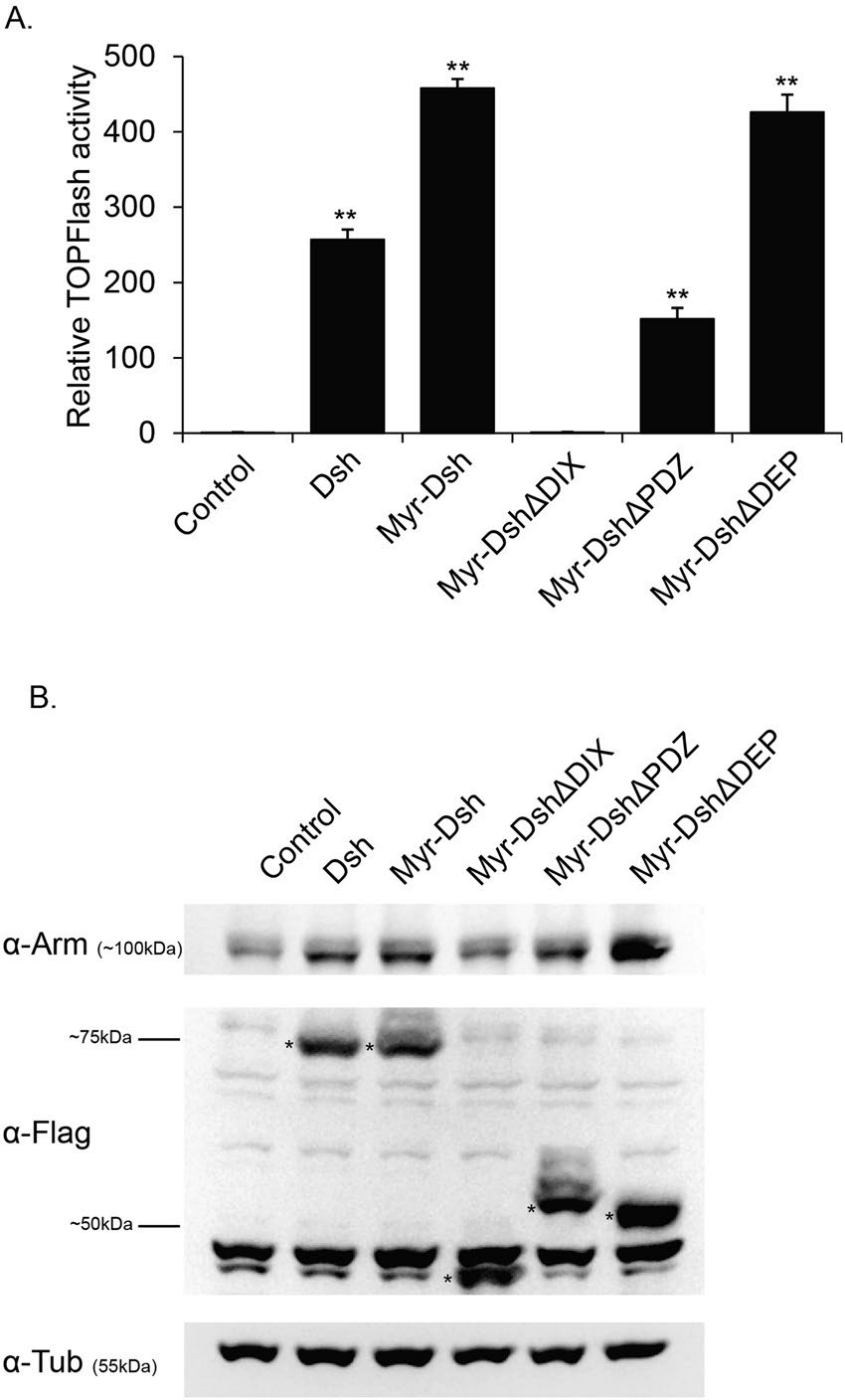
(**A**) Effects of Dsh deletion constructs on TOPflash reporter activity. S2R + cells were co-transfected with TOPflash reporter plasmid, Renilla luciferase-Pol III Vector and the indicated Dsh constructs. Mock transfected S2R + cells were treated as the baseline (control) for Wnt signaling activation. Results are representative of three independent experiments and the average of three replicates (mean ± SD). Statistical significance was tested using the Student’s t-test. **p < 0.01 relative to control. (**B**) Western blot comparing total Arm protein levels in cells transfected with the Flag tagged Dsh constructs indicated with *. α-tubulin was used as a loading control.

Previous work has led to a model where the DIX domain of Dsh is required for bringing Axin to the membrane, taking it away from the destruction complex^55^. Dsh transgenes lacking the DIX domain act as dominant negatives (Fig. 5A ^17^). In order to test our Dsh model further, we used a TopFlash assay, where the luciferase gene is attached to multimerized TCF binding sites to analyze functionality of Dsh variants in cell culture. We used untethered overexpressed Dsh in S2R + cells as the baseline for Wnt signaling activation and compared it to the various membrane-tethered Dsh constructs. We found that full length Myr-Dsh could activate the TopFlash promoter to a higher level than untethered Dsh (Fig. 6A; see Fig. 6B for expression levels). Deletion of the DIX domain in Myr-DshΔDIX did not activate the reporter. In contrast, Myr-DshΔDEP activated TopFlash reporter to a similar extent to full-length Myr-Dsh whereas Myr-DshΔPDZ activated to a somewhat lower extent (Fig. 6A), thus correlating with the *in vivo* results.

**Figure 7:**
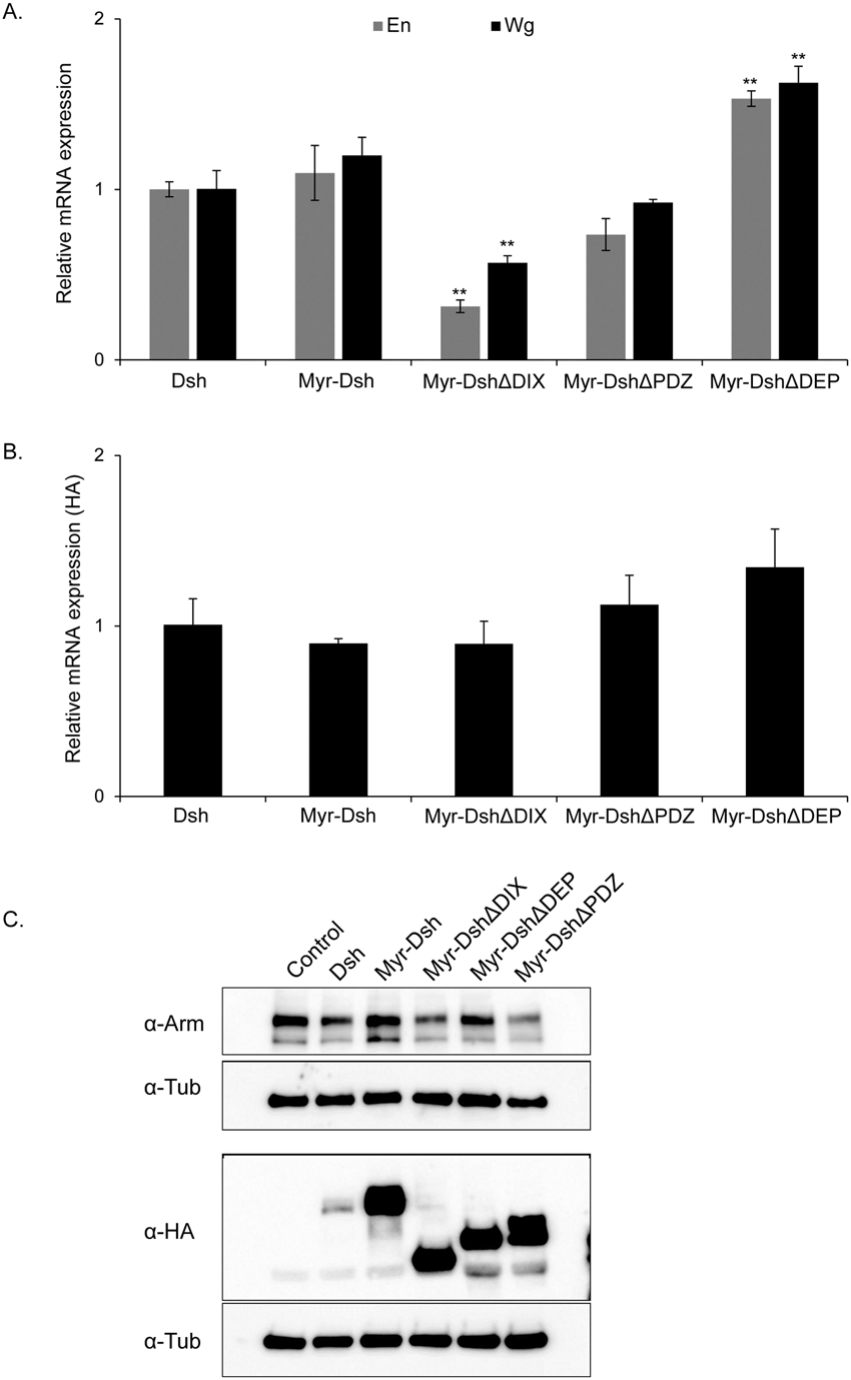
(**A**) Comparison of gene expression levels of *wg* & *en* in embryos expressing the indicated Dsh constructs. Reduced *wg* and *en* expression levels were observed in Myr-DshΔDIX expressing embryos compared to control embryos and embryos expressing Dsh. *p < *0.05*, **p < *0.01* relative to control. (**B**) Gene expression levels of HA Tag in embryos expressing the respective Dsh constructs. Significantly elevated expression of HA was detected in all transgenic lines compared to control. **p < *0.01*. (**C**) Western blot comparing total Arm protein levels in embryos expressing the various HA tagged myristoylated Dsh constructs.

We next looked at endogenous target genes downstream of Wnt signaling. Wnt signaling activates and maintains its own activity by activating *wg* and *en* transcription^45^. Using qRT-PCR, we therefore quantified *en* and *wg* transcript levels in embryos upon overexpression of the various Dsh mutant transgenes relative to wildtype Dsh (normalized to the housekeeping gene RpL32). We compared the various membrane-tethered Dsh constructs expressed in otherwise wild-type embryos (Fig. 7A; transgene expression levels are shown in 7B), and found that full length Myr-Dsh activated to a similar level to untethered Dsh. Consistent with the DN effect *in vivo* (Fig. 5A) the deletion Myr-DshΔDIX lowered the overall abundance of *wg* and *en*. Myr-DshΔPDZ showed an insignificant reduction in levels of *wg* and *en*, whereas Myr-DshΔDEP showed strong activation (Fig. 7A). We looked at Arm protein levels in the various conditions, and these correlated with the levels of Dsh activity with Myr-Dsh and Myr-DshΔDEP showing increased Arm protein (Fig. 7C). Taken together, these results support the overall activity levels of Dsh shown in the *in vivo* rescue and epistasis assays.

### Membrane localized Dsh is protected from degradation

As we looked at the levels of HA-tagged Dsh in embryos (Fig. 7B), we noticed consistently that the levels of cytoplasmic protein (Fig. 7C) were much lower than the expressed myristoylated forms (in spite of comparable activities). As these lines were made by phiC31 integration into identical sites, we expected similar levels of protein, but this was clearly not the case. We looked at the mRNA expression levels by qPCR, and observed that all Dsh forms were expressed at similar levels at the transcript level (see HA levels in Fig. 7B), but the protein levels were much lower for the un-tethered version of Dsh (Fig. 7C). This result shows that membrane localization may protect Dsh from degradation in the cytoplasm *in vivo* ^56^.

## Discussion

The identification of Dsh as an activator of the Wnt pathway and its placement in the pathway upstream of GSK3 and Arm led to a simple genetic description of the Wnt pathway. Yet years later, the molecular function of Dsh is still the subject of debate. Scaffold proteins such as Axin and Dsh perform complex roles in signaling by bringing several proteins into close proximity in different cellular compartments. Our work in this paper focuses on the role of membrane localization of Dsh, its relationship with the Wnt co-receptor Arr, and the domains utilized in the signaling process. We show that, genetically, the role of Arr is to localize Dsh to the membrane in response to Wnt, as Arr’s role in signaling is bypassed when Dsh is targeted to the membrane.

Our structure/function studies *in vivo* suggest that the DIX domain is absolutely required for signal activation, and further, its loss can cause a dominant negative effect. The DEP and PDZ domains are dispensable for canonical signaling only if Dsh is membrane tethered, but their absence decreases the effectiveness of membrane tethered Dsh in activating canonical signaling in the absence of endogenous Dsh and Arr, as shown phenotypically in our rescue assays of maternal-zygotic null alleles. We show that the PDZ domain is dispensable for Dsh function in canonical signaling as expression of Myr-DshΔPDZ rescues *dsh* (M/Z) embryos and activates strongly in otherwise wildtype embryos showing that the interaction of PDZ with Fz isn’t crucial if Dsh is at the membrane. But the PDZ domain does contribute to signaling as its absence weakens the activation in all our assays.

Although our results do not directly explain how the destruction complex is inactivated, they do point to a model of how the membrane-proximal activation complex or signalosome functions. Under normal signaling conditions, Dsh recruitment to the membrane is followed by GSK3/CK1 phosphorylation of sites on the cytoplasmic tail of Arr forming a binding site for Axin effectively disrupting the degradation complex. These sites work in conjunction with the Axin DAX/Dsh DIX interaction to form Wnt signal activating signalosomes^18, 23, 25–27, 31, 57, 58^. Our results suggest that localizing Dsh to the membrane is sufficient to remove Axin from the destruction complex, thereby blocking Arm degradation, especially as the membrane localized Dsh is protected from degradation. These findings do not necessarily distinguish between the several models for destruction complex inactivation, but in the absence of Arr, pseudo-substrate sites for inhibition of GSK3 cannot be formed at the membrane suggesting that this may not be the only way that GSK3 can be inhibited, and that the most likely mechanism of activation is the titration of Axin away from the cytoplasm.

We find that membrane localized Dsh accumulates to higher levels than normal Dsh through a post-translational mechanism. Previous studies have suggested that Dsh can be degraded through proteasomal degradation^56, 59^, but another recent finding suggests that the basolateral complex protein Discs Large protects Dsh from degradation^60^. This adds an interesting dimension to Dsh regulation as we have previously observed interactions between Wnt pathway components and apicobasal machinery^54, 61–63^. We attributed these effects to non-canonical signaling, but it could have effects on canonical signaling as well^5, 59, 64, 65^.

It was found that Dsh in vertebrate cell culture and Xenopus embryos shuttles between the cytoplasm and nucleus and that the ability to enter the nucleus is important for Dsh function specifically in canonical Wnt signaling^41^. It was suggested that nuclear Dsh might affect degradation of β-Catenin in the nucleus or indirectly in the cytoplasm. Our results showing that stabilized Arm is constitutively active in a M/Z *dsh* null background argues that any nuclear function of Dsh acts upstream of Arm and is not an additional, Arm-independent nuclear function of Dsh, a scenario that previously had not been excluded.

Taken together, we suggest that the membrane proximal activation complex brings together several proteins and enzymes – Fz, Arr, GSK3, CK1, Axin and Dsh. Formation of the complex leads to phosphorylation of Arr by GSK3 and CK1, creating binding sites for Axin brought to the membrane by the DIX domain of Dsh. Dsh is likely brought to the membrane through Fz binding to the PDZ and DEP domains and DEP binding to charged phospholipids. In our system, we can bypass the creation of Axin binding sites on Arr by directly tethering Dsh to the membrane.

## Materials and Methods

### Crosses and expression of UAS constructs

Maternally mutant eggs were generated by the dominant female sterile technique where balanced mutants are crossed to the dominant female sterile mutation *ovo* ^*D1*^ and recombination is induced using the FLP/FRT method in ovaries^47, 66, 67^. Oregon R was used as the wild-type strain. Please see Flybase for details on mutants used (flybase.bio.indiana.edu). Mutants used: *dsh* ^*V26(or 3)*^ and *arr* ^2^ ^53^. For mis-expression experiments, the ArmGAL4 2^nd^ chromosome and daGAL4 3^rd^ chromosome drivers were used. All X-chromosome mutants use FRT 101 except for *dsh* ^*V26*^ that has FRT 18E and second chromosome *arr* ^2^ mutants use the G13 FRT. The following crosses were conducted:

1. *arr* ^*2*^ FRTG13/*ovo* ^*D1*^ FRTG13; da-Gal4/ + females x *arr* ^*2*^/CyO-GFP; UAS-Dsh-3XHA
2. *arr* ^*2*^ FRTG13/*ovo* ^*D1*^ FRTG13; da-Gal4/ + females x *arr* ^*2*^/CyO-GFP; UAS-Myr-Dsh-3XHA
3. *arr* ^*2*^ FRTG13/*ovo* ^*D1*^ FRTG13; da-Gal4/ + females x *arr* ^*2*^/CyO-GFP; UAS-Myr-DshΔDIX-3XHA
4. *arr* ^*2*^ FRTG13/*ovo* ^*D1*^ FRTG13; da-Gal4/ + females x *arr* ^*2*^/CyO-GFP; UAS-Myr-DshΔPDZ-3XHA
5. *arr* ^*2*^ FRTG13/*ovo* ^*D1*^ FRTG13; da-Gal4/ + females x *arr* ^*2*^/CyO-GFP; UAS-Myr-DshΔDEP-3XHA
6. *y*, *dsh* ^*V26*^ FRT18E/*ovo* ^*D2*^ FRT18E; arm-Gal4/ + females x UAS-Dsh-3XHA
7. *y*, *dsh* ^*V26*^ FRT18E/*ovo* ^*D2*^ FRT18E; arm-Gal4/ + females x UAS-Myr-Dsh-3XHA
8. *y*, *dsh* ^*V26*^ FRT18E/*ovo* ^*D2*^ FRT18E; arm-Gal4/ + females x UAS- Myr-DshΔDIX-3XHA
9. *y*, *dsh* ^*V26*^ FRT18E/*ovo* ^*D2*^ FRT18E; arm-Gal4/ + females x UAS-Myr-DshΔPDZ-3XHA
10. *y*, *dsh* ^*V26*^ FRT18E/*ovo* ^*D2*^ FRT18E; arm-Gal4/ + females x UAS-Myr-DshΔDEP-3XHA
11. *y*, *dsh* ^*V26*^ FRT18E/*ovo* ^*D2*^ FRT18E; arm-Gal4/ + females x UAS-Arm^S56A^-2XHA
12. *arr* ^*2*^ FRTG13/*ovo* ^*D1*^ FRTG13; da-Gal4/ + females x *arr* ^*2*^/CyO-GFP; UAS-Myr-Arm^ΔN^-2XHA

X chromosomes were marked with the *yellow* ^*1*^ and *w* ^*1118*^ mutation and the CyO balancers were marked GFP to simplify analysis. For rescue of *dsh*, paternally rescued embryos were excluded as they were either y^+^ (fathers all had wildtype *y* alleles). For rescue of *arr*, paternally rescued embryos were excluded by selecting against a GFP balancer (genotype of fathers was with *arr* ^*2*^ over CyO-GFP). As mothers were heterozygous for the Gal4 source, maximal rescue is reflected by a drop of phenotype to 50% (only half of the embryos will express Gal4). For all crosses, more than 100 embryos were analyzed in multiple, separate experiments (n > 95).

### Transgenes and GAL4 driver lines

Two ubiquitous drivers were used for expression of transgenes: the weaker armadillo-GAL4 and the stronger daughterless-GAL4^68^. UAS constructs were made using Gateway recombination (Invitrogen). Myristoylated constructs were made by adding a sequence identical to the NH2 terminus of *src* (MGNKCCSKRQGTMAGNI) to the NH2 terminus of GSK-3 by PCR. This sequence has proven to be very effective for membrane targeting of Arm^16, 42, 43, 53^. The PCR products were then transferred by Gateway cloning (Invitrogen) into pUASg.attB with C-terminal 3XHA tag (A kind gift from J. Bischof and K. Basler, Zurich)^69^.

pCasp_dshDshΔDEP was made by amplifying the C-terminus of Dsh lacking the DEP domain with primers DshΔDEP_For_Xho (TAACCTCGAGGAGATCGTTAAGGCGATGACGAAGGAGCGCAATCCCAATCTGTTG) and DshΔDEP_rev_Xba (TAGTTCTAGAGTCGCGGCCGCTTTACAATACGTAATTAAATACGGA) and cloned as XhoI/XbaI fragment into pCasp_dshDsh_silentKpnSac_EGFP^51^. pCasp_dshDshΔDEP-CT was made by replacing the XhoI/XbaI fragment of pCasp_dshDsh_silentKpnSac_EGFP with annealed oligos DshΔDEP_CT_lower and upper (CTAGAGTCGCGGCCGCTTTACTTCGTCATCGCCTTAACGATCTCC; TCGAGGAGATCGTTAAGGCGATGACGAAGTAAAGCGGCCGCGACT). Transgenes were injected into either *w* ^*1118*^ (Casp constructs) or attP2 (Strain #8622) P[CaryP]attP2 68A4 by Rainbow Transgenics or BestGene Inc. (California)^70^. Relative rescue indices as described^51^. Briefly, *y* ^*1*^ *w* ^*1118*^ *f* ^*36-a*^ *dsh* ^*V26*^ */FM7 w* females were crossed to males carrying the appropriate transgenes. Offspring males with a transgene were counted. Rescued animals lack FM7 and are hemizygous for *f* ^*36-a*^ (to exclude X-chromosome non-disjunction events). The relative rescue index was counted as fractions of these males normalized to the average rescue efficiency of wild-type Dsh (note that wild-type Dsh constructs contain two silent point mutations^51^). To score activity of Dsh variants for non-canonical Wnt signaling, transgenic males were crossed to *dsh* ^*1*^ females and PCP defects in eye sections and wings were assessed in male offspring as described in^71, 72^.

### Antibodies and Immunofluorescence

Embryos were fixed with Heat-Methanol treatment^73^ or with heptane/4% formaldehyde in phosphate buffer (0.1 M NaPO4 pH 7.4)^16^. The antibodies used were: anti-Armadillo (mAb N2 7A1, Developmental Studies Hybridoma Bank developed under the auspices of the NICHD and maintained by The University of Iowa, Department of Biological Sciences, Iowa City, IA 52242), anti-HA (ratAb 3F10 and mouse 12CA5, Roche), rabbit anti-Armadillo^74^, phospho-tyrosine pY99 (Santa Cruz Biotechnology), anti-β-tubulin (E7, DSHB), and anti-FLAG (F9291, Sigma-Aldrich). Staining, detection and image processing as described in^75^.

### Western Blotting

Embryos were selected for fertilization and developmental stage, lysed in RIPA buffer (Cell Signaling Technology) with protease inhibitor cocktail (Roche), the extracts were separated on 4–20% gradient SDS-PAGE gel (Biorad), and blotted as described in^76^.

### TOPflash assay

TOPflash luciferase assays (TCF/LEF reporter assays) were performed to assess the effect of the Dsh deletion constructs on canonical Wnt-signalling. S2R + cells were co-transfected with dTF12 TOPflash reporter (TCF Reporter Plasmid; A kind gift from R. DasGupta, Singapore)^77^, Renilla luciferase-Pol III Vector (Promega) and the respective Dsh constructs using lipofectamine 3000 (ThermoFisher Scientific) according to the manufacturer’s instructions. Cell lysates were prepared 48 h after transfection and luciferase activity was measured using the Dual-Luciferase Reporter Assay System (Promega) according to the manufacturer’s instructions. The relative TOPflash luciferase activity was measured using the ratio of firefly/renilla luciferase activity and the data was presented as mean ± SD.

### RNA Extraction, cDNA Synthesis and qPCR

Total RNA was extracted for each experimental condition from 50ul of Drosophila embryos (collected 14–16hrs after deposition) using RNeasy Mini Kit (Qiagen) as per the manufacturer’s protocol. Total RNA concentration was measured using NanoDrop ND-2000 Spectrophotometer and the purity of the samples was determined by the OD ratios, A_260_/A_280_. One µg of total RNA was reverse transcribed in a 20 µl reaction volume using the QuantiTect reverse transcription kit (Qiagen) according to the manufacturer’s protocol. Gene specific primer sequences were obtained from Fly Primer Bank

(*en* forward primer, 5′-TCCGTGATCGGTGACATGAGT-3′;

*en* reverse primer, 5′-CGCCGACGTATCATCCACATC-3′;

*wg* forward primer, 5′-GACCCAGCGATCCACTCTAC-3′;

*wg* reverse primer, 5′-CGGCGATTTCTGAACTGGTGT-3′;

HA forward primer, 5′-GTTCCTGACTATGCGGGCTA-3′;

HA reverse primer, 5′-AGCGTAATCTGGAACGTCAT-3′;

*RpL32* forward primer, 5′-CCCAAGGGTATCGACAACAGA-3′;

*RpL32* reverse primer, 5′-CGATCTCGCCGCAGTAAAC-3′)^78^.

Quantitation of mRNA was performed using SYBR® Green Assay (Thermo Fisher Scientific) on the PikoReal™ Real-Time PCR System (Thermo Fisher Scientific) and a PCR product dissociation curve was generated to ensure specificity of amplification. *RpL32* was used as an endogenous control and relative quantitation was performed using relative quantification (2^−ΔΔCT^). Results were generated from 3 technical replicates for each mRNA. The average relative expression ± standard deviation (SD) was determined and two sample t-test was carried out to determine statistical significance.

## Electronic supplementary material

Supplementary Figure 1
