# Supplementary material for: Membrane Targeting of Disheveled Can Bypass the Need for Arrow/LRP5
Source: Sci Rep. 2017 Jul 31;7:6934. doi: 10.1038/s41598-017-04414-0 (PMC5537288; doi:10.1038/s41598-017-04414-0)

Supplementary Figure

**Membrane Targeting of Disheveled Can Bypass the Need for Arrow/LRP5**

Prameet Kaur^1^, Vanessa Yuk Man Lam^1^, Anirudh Gautam Mannava^2^, Jahnavi Suresh^1^, Andreas Jenny^3^, Nicholas S. Tolwinski^1,2^


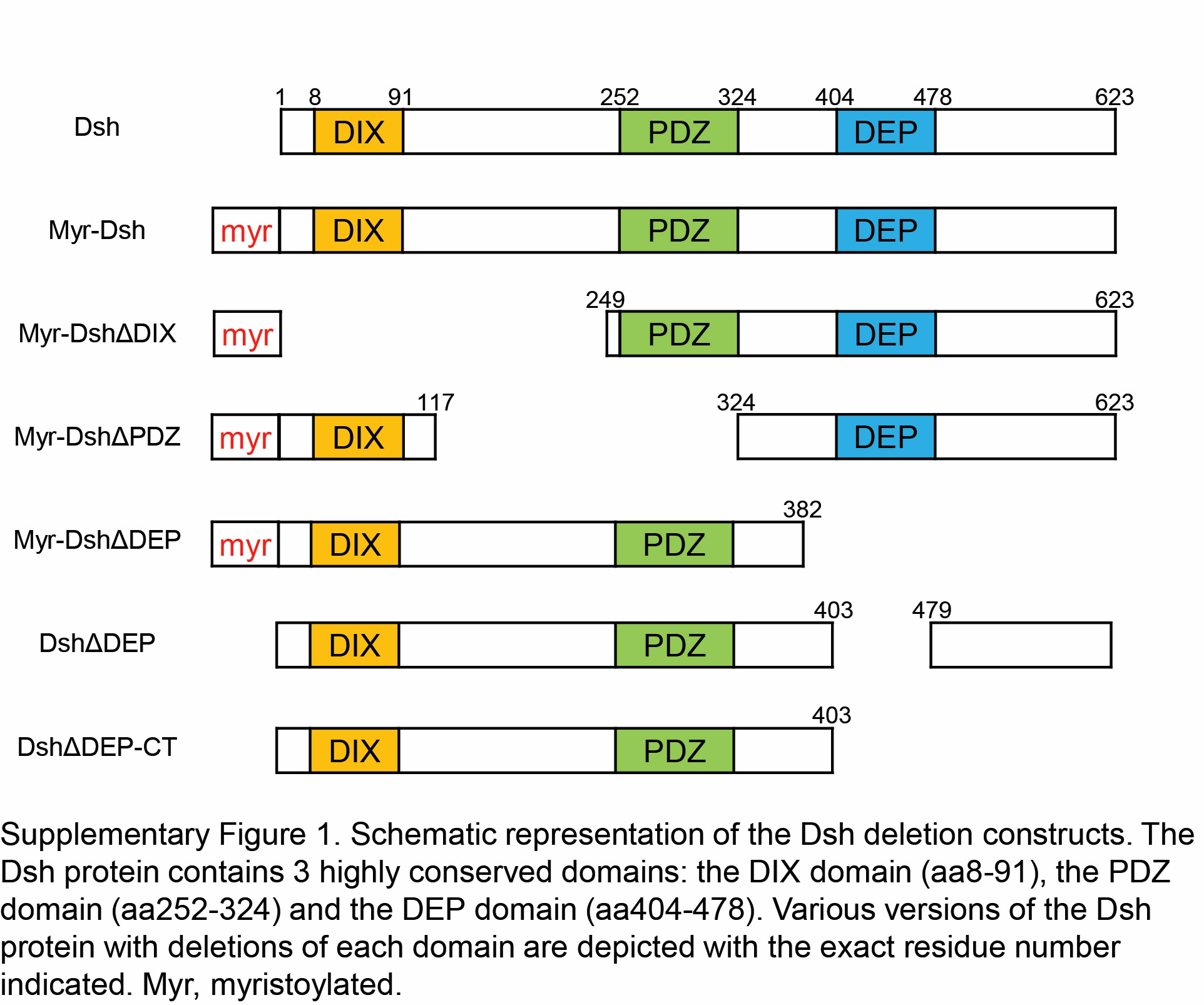

Supplement: Supplementary file 1 — Supplementary Figure 1 [file 41598_2017_4414_MOESM1_ESM.doc]
